# Methodological proposal for the inter-institutional management of wastes in health care centers in Uruguay

**DOI:** 10.1016/j.mex.2018.11.022

**Published:** 2018-12-14

**Authors:** Carolina Ramírez, Elizabeth Gonzalez

**Affiliations:** Universidad de la República, IMFIA - Facultad de Ingeniería, Rivera 2967 Apto 201, Montevideo, Uruguay

**Keywords:** Inter-institutional management, Waste management, Sanitary waste

## Abstract

This article focuses on defining a methodological proposal to appropriately address each of the aspects of the inter-institutional Management of Health Care Waste (RAS) that are generated within in the Health Care Centers (CAS) of the City of Montevideo. An application case based on the work experience of the Department of Environmental Engineering (DIA) team of the Institute of Fluid Mechanics and Environmental Engineering (IMFIA) of the Faculty of Engineering of the Universidad de la República, in a CAS of the city of Montevideo in which it contributed to significantly improve the inter-institutional management of RAS.

By focusing on the environmental issues related to the management of Health Care Waste and specifically in Uruguay, it can be said that between the starting point of the execution of these studies (2007) and the present, there have not been any developments made by this team, other systematic studies related to the realization of diagnoses of the situation of the handling of Health Care Waste, or determination of the rates of generation in National Health Care Centers. In fact, the generation rates known until then corresponded to bibliographic data, of which most of the time there is no clear information on how they were obtained.

It is here, where the importance of the approach in this methodology lies, since the qualitative as well as the quantitative information obtained from the studies developed corresponds to the genuine data of Health Care Centers of Uruguay.

The methodology defined from these studies is the result of the analysis of management systems corresponding to our reality, which guarantees that the methodology is supported by real theoretical and practical foundations, ensuring the functionality and efficiency of this methodology.

## Graphical abstract

**Figure:**
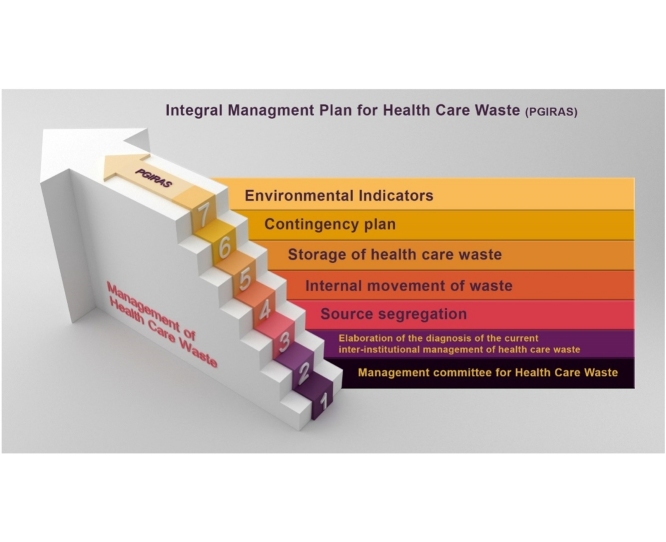

## Introduction

The definition of health-care wastes (HCW), or medical Wastes (MW), can vary significantly among countries in the European Union, MW are the wastes of chapter 18 of the European Waste Catalogue [1] which are defined as the wastes from human or animal health-care and/or related research. The World Health Organization (WHO) designates MW as the wastes generated by health-care activities that can include a wide range of materials, such as used needles and syringes, soiled dressings, body parts, diagnostic samples, blood, chemicals, pharmaceuticals, medical devices and radioactive materials. Variable definitions of MW exist in other parts of the world too [2]. In addition, it is not often clear whether household type wastes (non-hazardous wastes) are also included in the medical wastes. For example, in Jordan [3], grouped medical Wastes into: (i) pathological waste, (ii) sharps and (iii) infectious wastes.

In Taiwan, Cheng et al. (2009) [4] and Cheng et al. (2010) classified medical wastes into infectious wastes and general medical wastes without clearly specifying the contents of these two categories. In Turkey, the Wastes generated by health-care facilities (HCF) are grouped into municipal solid wastes, hazardous wastes, radioactive wastes, and medical wastes. The latter group is further classified into infectious wastes, pathological wastes and sharp objects. In China [5], medical wastes are grouped into tissues, infectious wastes, sharps, chemical wastes and medicine wastes.

In recent years, researchers of waste management focus on waste assessment and recommend many methods and tools for it, such as life cycle assessment ([6], Guilherme Marcelo Zanghelinia et al., 2014), impact categorical groups [7], standardized management systems [8], and environmental management accounting [9]. These methods and tools provide the references for medical waste-generation, but their applications in hospital waste management need to be verified in practice and their limitations need to be modified [10,7].

The priority of the Health Care Centers (CAS) is patient care and, consequently, its policy has traditionally been oriented towards the benefit of the patient's health and well-being, which has diminished importance to environmental problems. Currently in Latin America there has been confirmed growing interest regarding the management of Health Care Waste, which has triggered conducting various studies to determine the rate of solid waste generation in CAS, as well as the performance and diagnoses of the situation of the management of wastes. However, the comparative analysis of the results obtained in these studies should take into account that the methodologies used in each case, and even the basic definitions adopted regarding Health Care Waste, were different. In addition, in the countries of the region, the scarcity of environmental statistics is notorious, and the situation of these countries is heterogeneous, both in terms of the capacity to produce statistics and the quality of the resulting information. In order to progressively overcome this situation and to advance as a region towards the production and harmonization of environmental statistics, it is necessary to consolidate institutions and develop national technical capacities.

The management of Health Care Waste (RAS) presents potential risks to the health and safety of those working in the CAS and for the general population; this has been the principle motivation in Uruguay to begin studying and working in the management of Health Care Waste. In effect, in Uruguay, the comprehensive management of RAS has been regulated since 1999 and is currently governed by Decree 586/009 [11]. Article 23 of this Decree establishes that the CAS must have a Comprehensive Health Care Waste Management Plan (PGIRAS), approved by the Ministry of Public Health and the Ministry of Housing, Spatial Planning and the Environment. The elaboration of the PGIRAS is structured based on two general components: internal management component and external management component. In the specific case of the city of Montevideo, there are currently no systematic studies concerning the Management of Health Care Waste (diagnosis of the situation of Health Care Waste management or determination of the rates of generation of these wastes), which leads to the generation rates to be used correspond to bibliographic data, most of which do not have clear information on how they have been obtained.

## Material and methodology

### Previous experience

In recent years, DIA-IMFIA has carried out several projects to improve the inter-institutional management of RAS in Health Care Centers in the city of Montevideo. We analyzed the inter-institutional waste management system of a public CAS, in which a detailed diagnosis of the management system was carried out, from which modifications were proposed and implemented that resulted in improvements in management.

The CAS in which the study was conducted is a tertiary hospital care of adults with acute diseases, which has outsourced cleaning services. The personnel of this company carries out the collection and transport of waste within the CAS and has the responsibility of providing the bags for waste. Currently the CAS manages its waste so that those similar to urban zones are removed and transported by a private company to the final municipal disposal site, while the contaminated waste is transferred, received, treated and disposed of by another private company, which has the corresponding authorization.

#### Workplan

Rezzano et al. [12] established the following specific objectives to be achieved with the analysis of the health care management system:

• Have a detailed diagnosis of the waste management in the CAS.
• Generate management protocols to enhance and formalize the waste management procedures at different stages.
• To implement the proposed protocols in the CAS, using a training program for CAS staff in order to provide the different members with the necessary knowledge to do so.

In order to meet each of the particular objectives proposed, they were grouped into defined work stages as follows [13]:

1 Diagnosis of the initial situation
  In order to make a diagnosis in relation to waste management as it was carried out in the CAS, initially the background and existing documentation related to the subject and the services that work in the Health Care Center must be identified. In addition, the location of the waste containers, capacity, type of containers and bags used in each room, collection methods and practices, internal transport circuits, characteristics and location of transitional and final storage. Based on this information, which includes the types of RAS that are generated and the characteristics of the bags and containers used in the different areas of the CAS, maps of the initial situation were drawn up; it is clear and the results of the diagnosis can be easily understood, especially regarding the characteristics and location of containers for solid waste and intermediate deposits, as well as the circulation circuits of the different types of waste [14].
2 Evaluation of sanitary waste retrieval rates
  The amount of waste that is generated in a Health Care Center is a function of the various activities that take place in it and will depend on the number of medical services offered in the establishment, the degree of complexity of the care provided, the size of the Health Care Center, and the proportion of outpatients and staff. Therefore it is not easy to establish simple relations that allow one to estimate the amount of Health Care Waste in use, due to such diversity of factors. This has led, in most cases, to relate the average amount of solid waste generated daily to the number of CAS beds, thus obtaining figures that; although subject to a certain degree of imprecision, are easier to manage and apply. Table 1.

**Table 1:** Total, generation of ras according to region.

Region	Kg/bed /day
North America	7–10
Latin America	2,6–3,8
Western Europe	3–10
Middle East	1,4–2
Asia	1,3–3
  In Latin America and the Caribbean the scarcity of environmental statistics is notorious and the situation within the countries is heterogeneous, both in terms of the capacity to produce the statistics and the quality of the resulting information [15]. More specifically, in the city of Montevideo there are not many systematic studies regarding the rates of generation of Health Care Waste, which means that the generation rates used correspond to bibliographic data, of which, there is no clear information on how the data was obtained. However, the Director of Solid Waste Montevideo and Metropolitan Area (AMM) (2005), states that waste generation Care contaminated Health of different health centers in the area in question depends on the activities in each of Health Care Centers and varies between 0.1 and 1.6 kg/bed/day.
  For the development of this methodological proposal, we will work with historical data corresponding to the period between 2008 and 2011, referring to rates of removal of contaminated, common and recyclable sanitary waste. These were provided by the staff of the Department of Hygiene and Cleaning of the CAS. The data available are: daily removal of contaminated and common waste, and monthly data of recyclable waste removal. Contaminated waste data are quantified in terrines; the common and recyclable, in kilograms. Although it is usual to work with waste generation rates, the characteristics of the available data led to the need to work with withdrawal rates, in order to avoid introducing uncertainty or error factors in order to consider hypotheses for relating waste quantities withdrawn and generated. In fact, since there was no daily weighing of waste in the CAS, and because there was no withdrawal service on Saturdays or public holidays, it was necessary to manage the information of the available records: in the case of contaminated waste, the number of terrines withdrawn each day, since the contract of withdrawal was expressed of that form.
  To calculate a rate of generation or withdrawal rate, in kilos of medical waste per day, it was necessary to assume an average weight of waste within the terrine. For this, it is assumed that the weight of waste per terrine can be estimated at 15 kg, a figure that emerges from a survey conducted in 2008 by the Department of Environmental Hygiene Hospital Center. Considering a terrine volume at 120 L, results in a density of 125 kg / m3, which corresponds, according to the experience of the team, to contaminated waste compaction degree.
  Taking the available data and punctual point values available in the CAS, and the quantities of waste removed per day in kg/day were obtained. These are presented in Fig. 1, for all days on which data are available. The absolute peak of withdrawal in the period was 3010 kg/day which corresponded to December 26, 2008, the Friday after the Christmas holiday. On that day 570 kg of contaminated waste and 2440 kg of common waste were removed; both values correspond to two days of generation, because the previous day there was no withdrawal of any type of waste.

**Fig. 1:**
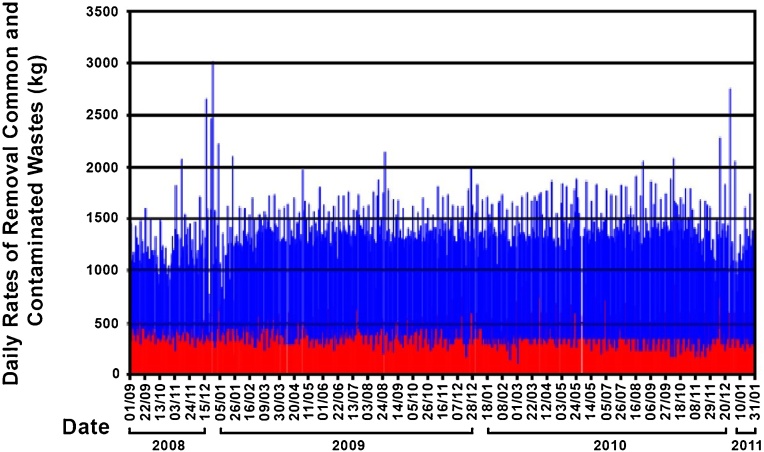
Daily rates of removal of common and sanitary waste in the period September 2008–February 2011. The blue bars correspond to common waste and the red bars to sanitary waste.
3 Design and implementation of a training program
  The program is designed to serve three different publics with different responsibilities but also with distant positions in relation to their knowledge of the subject: service personnel, health care personnel and medical professionals working in the areas of the CAS. The main results of the training programs are written materials for each level of training, which emphasize the specific responsibilities of the different personnel involved are generated.
4 Development of management protocols
  The work team developed and presented a general protocol for inter-institutional waste management, which defines explicit guidelines for the different stages of the varried solid waste types, including segregation, packaging and identification, internal collection and final storage, seeking the best organization to ensure the safety of service personnel, care staff, patients and companions. Waste management protocols were also proposed for each CAS generating service and management protocols for some types of Health Care Waste considered special, such as: out of date drugs, Anatomopathological waste, cytostatic waste, mercury-containing waste, recyclable waste, chemical wastes and radioactive waste.

### Methodological Proposal

This methodological proposal gives the Centers of Attention to Health of the city of Montevideo, the possibility of working in a schematic way within the inter-institutional management of the waste of Health Care. The fulfillment of each of the activities proposed in the PGIRAS content guarantees both: the service personnel and the health care personnel of the Health Care Centers of the city of Montevideo that the waste generated will be managed in the correct manner.

The elaboration of this methodological proposal also allows it to improve the execution of those activities included in the management of RAS in which shortcomings were identified, establishing some steps to follow to ensure that the incorrect practices are eliminated. In addition, new management procedures are established, referring to aspects that were not incorporated in the management of Health Care Waste in CAS.

The main aspect developed in this proposal is the presentation of the main points that must be developed within the Content of the Integral Management Plan for Health Care Waste (PGIRAS). In addition, a general protocol of inter-institutional waste management and management protocols for some types of RAS, such as recyclable wastes (paperboard, clean paper, plastics and glass) and some RAS considered to be special, such as out of date drugs (or dugs not suitable for use), Anatomopathological wastes cytostatic wastes, wastes containing mercury, wastes of chemicals, and radioactive waste. Also, training guides are presented for the personnel of the Health Care Centers, more specifically for the Personnel of Service and Personnel Assistance, in these guides the goal is to outline the main aspects that must be taken into account when it comes to training both the assistance and service personnel of the CASs; with the base being, informed practice for the best performance of their daily work. Situations derived from bad practices are not considered, in the understanding that they are abolished, nor are there guidelines for action in cases of contingency.

Once the Health Care Waste leaves the CAS, they are collected by the companies providing the service, it is assumed that the internal or inter-institutional management of this waste, i.e.: all activities carried out with these within the CAS, have been correctly executed, i.e.: the treatment plant were the Health Care Waste will be sent when leaving the CAS depends on the management. For this reason, this methodological proposal defines the aspects necessary to generate an Inter-Institutional Management System for Health Care Waste. The following hypotheses were taken regarding the management of RAS:

#### Contents of the integral management plan for health care waste - (PGIRAS)

**Fig. 2:**
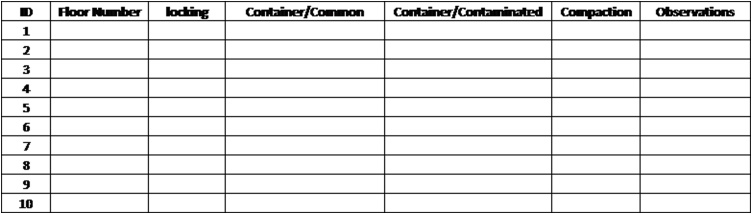
Data collection sheet - Intermediate Deposits.
- *Internal Transportation*
  During the execution of the diagnosis of Intrainstitutional Management of Health Care Wastes from the CAS, some aspects related to the transportation of the waste from the intermediate deposits to the final storage tank of the CAS must be identified: officials who carry out the transportation from the intermediate deposits located on the different levels of the CAS to the final storage tank, location of the necessary protection elements to carry out this task, characteristics and capacity of the carts used to transport the waste, infrastructure used within the CAS to transfer the waste, specifically if ladders are needed, if exclusive forklifts are available to carry out this task, collection circuits identified in the CAS. They must be presented graphically in the architectural plans of the CAS.
- *Final storage*
  All Health Care Waste generated in the CAS, once collected from the intermediate tanks or the service where they were generated, must be transferred to the CAS final storage tank. There different types of waste are stored until they are withdrawn by the companies contracted for this purpose.
- *Special waste management*
  The diagnosis of Inter-Institutional Management must include the generation and description of the management that is given to the special wastes generated in the CAS (oncological, out of date drugs, chemical substances, used batteries, light tubes) in terms of services in the which they are generated, packaged and collected and stored.
- *Generation of waste by sector*
  As part of the diagnosis of Health Care Waste management, a weighing system should be carried out to determine the common and contaminated waste generation rates within each of the main services of the CAS. This activity will consist of the systematic execution of waste weights during the period of diagnosis and must be carried out by the collectors of the company responsible for the cleaning. (The procedure defined for carrying out the weights is discussed below.) It is important to clarify that, although this procedure is used to evaluate the rates of generation of waste by sector during the diagnosis, the system of weights and registration are carried out on a permanent basis and is incorporated as a routine task to those already assigned to the collectors.

- ***Management committee for Health Care Waste***
  For the design and execution of the PGIRAS, the first step, a Health Management Waste Management Committee should be set up within the CAS, which will be made up of the staff of the institution, whose positions are related to the management of Health Care Waste and who will be the managers and coordinators of the PGIRAS.
  At the time of forming this committee the following aspects should be considered:
  The Health Care Waste Management Committee will be made up of: CAS General Director, Administrative Director, Financial Director, a CAS official who, because of their knowledge about the subject of Health Care waste, is qualified to lead the design and correct implementation of the PGIRAS, head of General Services, Coordinator of Occupational Health, an official of the technical staff (doctor or nurse).
  Previously established Infection Committees can be the basis for forming the Health Care Waste Management Committee, adapting its structure to the requirements of the Intrainstitutional Management of Health Care Waste.
  The Health Care Waste Management Committee must meet at least once a month to evaluate the implementation of the PGIRAS and take pertinent measures to ensure compliance. In addition, special meetings should be held when any of the members of the Committee deems it appropriate, always keeping records of the topics dealt with in the meeting. The main responsibilities that the Health Care Waste Management Committee must fulfill are the following: to carry out the Diagnostics of the inter-institutional management of Health Care Waste, to design the Integral Management Plan for Health Care Waste (PGIRAS), design the functional structure corresponding to PGIRAS and assign responsibilities to carry it out, define and establish coordination mechanisms, propose the budget of the plan, ensure the execution of the plan (train, control and sanction), prepare reports and internal reports.
  Once the Health Care Waste Management Committee is formed, the PGIRAS must include: diagnosis of the inter-institutional management of Health Care Waste, segregation at the source, selection and implementation of a system of treatment and disposal of Health Care Waste, Internal Waste Movement, Intermediate and central storage, preparation of the Risk Prevention Plan, preparation of the Contingency Plan, definition of inter-institutional management indicators, preparation of the Training Plan, reporting and internal reports to the corresponding environmental control authorities, periodic review and continuous improvement of programs and activities.
- ***Elaboration of the diagnosis of the current inter-institutional management of health care waste***
  To carry out a diagnosis of the situation within management of Health Care Waste in a CAS, the qualitative and quantitative characterization of the waste generated in the different areas of the institution must be carried out. For this, the exact location of the waste containers should noted, collection practices, internal transport circuits, characteristics and location of transitional and final storage areas in the different sectors. Once the sources of waste generation are identified, it is necessary to estimate the quantities and types of waste generated daily, and then to register them on forms designed for this purpose, it is convenient to refer to plans or diagrams of the plant, which indicate the location, typology and capacity of waste containers at generation sites, buffer sites and the flow of contaminated and non-contaminated waste for each of the CAS sectors.
- *Types of Health Care Waste Generated by Service*
  At this stage of diagnosis, the different types of waste generated in each of the CAS's administrative and care services must be determined.
- *Characteristics of the bags and containers used*
  We proceed to determine the characteristics of the bags and containers used in each of the services of the CAS, to collect Health Care Waste at the time of its generation and according to the category in which they belong.
- *Placement of containers and bags in the building*
  The location of the containers is presented graphically in the architectural plans of the CAS, which must be provided by CAS officials. There must be plans of each of the levels of the CAS. The information presented in the plans must correspond faithfully to the survey carried out during the diagnostic tasks. The current location, typology and capacity of the waste receptacles, the intermediate or final storage sites, as deemed fit; the route of the contaminated and uncontaminated waste from the generation points to the corresponding storage sites, and finally, to the retirement points.
- *Internal Collection*
  This stage of the diagnosis consists of defining the following aspects of the collection of RAS, from the primary collection vessel to the corresponding intermediate reservoir: personnel in charge of the task, frequency of collection, closure of bags to their transfer, and traceability.
- *Intermediate deposits*
  In the execution of the diagnosis, the precise location of the intermediate deposits within each level of the CAS must also be identified. In order to facilitate the process of surveying intermediate deposits in CAS and systematize the information corresponding to the main characteristics of these collection sites, it is recommended to use a survey form similar to the one presented in the Fig. 1. González et al. [14] (Fig. 2):

#### Procedure

Since it is sought to quantify the waste generated by generation sector, the staff of the cleaning company will be asked not to collect or dispose of waste from different sectors in the same car or tank until they are weighed.

The collector of the corresponding shift must transport the waste directly to the weighing site, without mixing it with waste generated in another sector. This is done with both the common and contaminated waste streams.

The weighing is done in the final storage tank corresponding to the type of waste in question, where a scale must be available to carry out the weighing.

• *Training and education program*
  The training and education program contemplates the training strategies and methodologies necessary for compliance with the PGIRAS, which include theoretical training, practice and training.
  In order to design training and education programs to be implemented in a CAS, it is essential to clearly define the aspects of the Inter-Institutional Management of Health Care Waste in which work is needed. For this purpose, the design of a Risk Prevention Plan is proposed.
• *Training programs*
  The development of training programs conceived from a "lifelong education" perspective; and it is key to achieving the objectives of the PGIRAS of the CAS, especially in relation to the correct segregation of Health Care Waste and the reduction of risks during the handling of Contaminated Waste.
  *Levels of training:* It is necessary to orient the tasks, with different methodologies and contents to three groups:
• Patients and companions.
• Assistance staff (nursing assistants, university nurses, doctors).
• Service personnel (CAS and cleaning company officials).
• ***Source segregation***
  For the correct segregation of Health Care Waste, the containers will be located in each of the areas and services of the Health Care Center (CAS), in the quantities needed according to the type and amount of waste generated. Based on the results of the situation diagnosis of the CAS Inter-institutional Management, management protocols should be developed for each of its sectors. The protocols include explicit segregation guidelines to the sites where the containers should be located for each type of waste, guidelines for internal transport and storage, in order to ensure the safety of service personnel, care staff, patients and companions.
• ***Internal movement of waste***
  Each CAS should define the times of collection and transportation of waste, including routes and frequencies to avoid interference with other activities.
• ***Storage of health care waste***
  According to the complexity level and the size of the Health Care Centers, the following types of inter-institutional storage are established: initial or primary storage, temporary or secondary storage, final or tertiary storage.
• ***Contingency plan***
  The Contingency Plan is an integral part of the Health Care Waste Management Plan (PGIRAS) and should contemplate the measures for emergency situations associated with the management of SAN. Whose possible occurrence is sensible to consider, although its probability is not high.
  The main situations that must be considered in the contingency management plan that is developed; within the framework of the integral waste management plan within the Health Care Centers are:
• Spill of contaminated waste inside Hospital facilities.
• Occurrence of fires.
  The occurrence of major accidents with risk to human life is not considered due to the imminent proximity of assistance in the event of an event of this nature.
• ***Environmental Indicators***
  Environmental indicators allow the collection of information related to data of different a nature, and presented in a summarized way for rapid understanding and evaluation, in order to be used in decision making; for this it is necessary that they be comparable indicators, that aim at goals and that reflect the environmental performance with the maximum fidelity possible. The environmental indicators can be classified in the following categories:
• Environmental quality indicators: they provide information on the external environmental situation to the CAS at the local or global level. For this reason, the application of this type of environmental indicator does not correspond in this proposal, since only a reference is made to the Inter-Institutional Management of Health Care Waste.
• Indicators of environmental performance: they are used as tools of control and registry of improvements in environmental management. These indicators are based on the planning, control and monitoring of environmental impact and evaluate the efficiency and environmental performance of operations or processes within the organization. For example: Total common health care waste (kg)/Total health care waste (kg), Cost of health care waste removal (bed)/Cost of health care waste removal (liters), Recyclable health care wastes (kg)/Common health care wastes (kg), Fine glass (grams)/Sharp health care wastes (grams).
• Environmental management indicators: they are used to perform management control and evaluate the organization's efforts to improve its environmental performance, but does not provide information on the performance of the organization. For example: kg of Special Health Care Wastes in Common Wastes/Common Healthcare Wastes, Kg of Common Health Care Wastes in Special Wastes/kg of Special Healthcare Wastes, Drugs Returned to time / Drugs purchased, Drugs to dispose/Drugs purchased.

## Additional information

Some of the improvement opportunities identified are outlined below and it is established and how the situation related to the management of RAS that originates each opportunity for improvement is currently being addressed in the management activities carried out in Montevideo by CAS. Having been studied by this team. It also defines how it has proposed to addressed the issues in the CAS in accordance with the provisions of Decree 586/009.

1 Standardization of containers, according to the different categories of waste generated, both in the types of containers and in the identification of the colors
2 What was being done: During the conduct of the basic studies, prior to the PDRS, it was determined that the containers used to place the contaminated RAS prior to their transfer to storage sites did not have the same levels of safety in all CAS.
3 What was proposed: The provisions of Article 9 of Decree No. 586/009 must be fully complied with. For the correct segregation of RAS it is proposed to place containers in each of the areas and services of the CAS, in the quantities necessary according to the type and amount of waste generated.
4 Each of the CAS must have personnel responsible for the management of RAS in general.
5 What was being done: In general, large CAS assigned this task to a person who, in general, does not exclusively handle the internal management of RAS.
6 What was proposed: Decree No. 586/009 in Chapter III, Article 6, establishes that CAS must implement Sanitary Waste Management Committees, which will be controlled by the competent authority. This Committee should be made up of personnel of the institution whose positions are related to the management of Health Care Waste and who will be the managers and coordinators of the design and implementation of the PGIRAS.
7 Individuals who decide the destination of the waste at the time of placing it in containers that have been installed in a timely manner, must be sufficiently qualified to discriminate according to their characteristics, being aware of the associated risks.
8 What was being done: It was found that those responsible for the internal management of RAS had not defined specific training programs for CAS staff who; because of their activities, were in contact with RAS.
9 What was proposed: The developed PGIRAS indicates the need to have training instructions for CAS personnel involved in the management of RAS, within the framework of a continuing education program. Three training manuals are presented, the content of which depends on the CAS staff who, according to the group to which the training program is directed: service personnel, health care personnel and medical professionals working in the CAS.
10 The basis for successful training is the training plan prepared by each CAS internally. This plan must be prepared by the RAS Management Committee, together with the management of the center or be approved by it. Training must combine two aspects: be in line with the previous level of training of those who will receive it; be in agreement with the role that those individuals have in the institution. The elements that a training plan must contain, according to what is established in the PDRS are: definition of groups of people to be trained, list of people already trained, records of training instances, contents of training programs for each of the groups.
11 It is recommended that each CAS should aim at a maximum generation between 15 and 25% of contaminated RAS with respect to the total of RAS generated in the center. For CAS with a rate greater than 20% of contaminated RAS, it is advisable to improve the internal classification of their RAS.
12 What was being done: some situations detected in CAS with high percentages of contaminated RAS, which favor the inclusion of common RAS in the stream of contaminated RAS, are in the incorrect location or identification of vessels, negligence of those responsible for classification, poor or insufficient training of personnel, insufficient controls by the generator and the authorities.
13 What was proposed: The diagnosis of the situation of the current management of RAS in a CAS consists of the qualitative and quantitative characterization of the waste generated in the different areas of the institution. For this, the location of the waste containers, collection practices, internal transport circuits, characteristics, should be detailed. Once the sources of waste generation are identified, the quantities and types of waste generated daily must be estimated, and then registered in the corresponding forms, and it is convenient to reference the generation sites using plans or plant diagrams, which indicate the current disposal, typology and capacity of the waste containers, intermediate storage sites and the flow of contaminated and non-contaminated waste, for each of the levels of the CAS. This will allow to define with certainty the capacity and quantity of containers necessary for each type of RAS that are generated in the different areas of the CA.
